# Prediction of anemia at delivery

**DOI:** 10.1038/s41598-021-85622-7

**Published:** 2021-03-18

**Authors:** Enav Yefet, Avishag Yossef, Zohar Nachum

**Affiliations:** 1 Department of Obstetrics and Gynecology, Emek Medical Center, Yitzhak Rabin Boulevard 21, 1834111 Afula, Israel; 2 Department of Obstetrics and Gynecology, Baruch Padeh Medical Center Poriya, Tiberias, Israel; 3 Azrieli Faculty of Medicine, Bar-Ilan University, Safed, Israel; 4 Rappaport Faculty of Medicine, Technion, Haifa, Israel

**Keywords:** Epidemiology, Medical research, Risk factors, Health care, Diagnosis, Disease prevention

## Abstract

We aimed to assess risk factors for anemia at delivery by conducting a secondary analysis of a prospective cohort study database including 1527 women who delivered vaginally ≥ 36 gestational weeks. Anemia (Hemoglobin (Hb) < 10.5 g/dL) was assessed at delivery. A complete blood count results during pregnancy as well as maternal and obstetrical characteristics were collected. The primary endpoint was to determine the Hb cutoff between 24 and 30 gestational weeks that is predictive of anemia at delivery by using the area under the curve (AUC) of the receiver operating characteristic curve. Independent risk factors for anemia at delivery were assessed using stepwise multivariable logistic regression. Hb and infrequent iron supplement treatment were independent risk factors for anemia at delivery (OR 0.3 95%CI [0.2–0.4] and OR 2.4 95%CI [1.2–4.8], respectively; C statistics 83%). Hb 10.6 g/dL was an accurate cutoff to predict anemia at delivery (AUC 80% 95%CI 75–84%; sensitivity 75% and specificity 74%). Iron supplement was beneficial to prevent anemia regardless of Hb value. Altogether, Hb should be routinely tested between 24 and 30 gestational weeks to screen for anemia. A flow chart for anemia screening and treatment during pregnancy is proposed in the manuscript.

Trial registration: ClinicalTrials.gov Identifier: NCT02434653.

## Introduction

Anemia during pregnancy, defined as hemoglobin (Hb) < 10.5–11 g/dL, is experienced by 10–40% of women^1,2^. Short and long-term complications include dyspnea and fatigue, decreased functional capacity, increased risk for maternal infections, impaired quality of life, poor cognitive performance and emotional instability. Anemia at delivery is a risk factor for postpartum anemia, which is associated with increased risk for blood transfusion, postpartum depression, impaired mother–child interactions, and increased mortality and has important potential implications for the future neuro‐development of the infant^1,3–11^.

There is insufficient data to determine the most appropriate time for anemia screening in pregnancy and follow-up. Guidelines regarding treatment of anemia based on Hb levels are also unclear; the American College of Obstetricians and Gynecologists (ACOG) recommends screening all pregnant women for anemia, but provides no exact timing and schedule^2^. The National Institute for Health and Care Excellence (NICE) guidelines advise testing at the beginning of pregnancy and at 28 weeks, yet, it was mentioned that evidence to support this recommendation is lacking^1,12,13^.

In the present study, we assessed Hb during pregnancy and other maternal characteristics as predictors for anemia at delivery.

## Methods

### Design

This was a secondary analysis of data collected in a prospective cohort trial which assessed the efficacy of a routine screening protocol for postpartum anemia diagnosis and treatment of women in the maternity ward who delivered vaginally. The study evaluated two protocols for postpartum anemia detection^14^.

The study was conducted between June 29, 2015 and January 27, 2016, at the university-affiliated Emek Medical Center in Israel (ClinicalTrials.gov Identifier: NCT02434653, date of registration: 28/04/2015). This study was authorized by the local review board of the Emek Medical Center (EMC 112-14) and was performed in accordance with relevant guidelines and regulations of the institutional review board. Participants provided written informed consent.

Women who intended to or eventually delivered vaginally (spontaneous or by vacuum extraction) were tested for eligibility at the labor and delivery, maternal fetal medicine, or maternity wards. Inclusion criteria were women above 18 years of age who delivered vaginally ≥ 36 gestational weeks. Women who had known allergy to iron sucrose or pre-eclampsia with severe features, were not eligible to participate in the study.

Anemia at delivery was defined as Hb < 10.5 g/dL at complete blood count (CBC) taken prior to or immediately after delivery^15^.

CBC test results during pregnancy and at delivery were either obtained at the obstetric department or collected from the database of the Emek Medical Center hematology laboratory, where most of the CBC tests of the area are performed. Data regarding health status, iron supplement use during pregnancy and vegetarianism, were collected using questionnaires (in Hebrew and Arabic) that were given after delivery, during admission to the maternity ward. Women were asked to rank, on a scale of 0 (least disturbing) to 10 (most disturbing), the following parameters for the week before delivery: fatigue, dizziness, palpitations, shortness of breath and pre-syncope (sensation of blurred vision or about to faint). For analysis of the questionnaires, the mean of 5 questions regarding anemia symptoms (fatigue, dizziness, palpitations and shortness of breath) were combined into one parameter after calculating the Cronbach Coefficient Alpha > 0.7, which suggests that these questions are well correlated and can be combined. For simplicity, we called this score "anemia-related symptoms (ARS) score", although these symptoms can be reported by non-anemic persons as well.

### Choosing the time range during pregnancy in which Hb will be tested as a predictor for anemia at delivery

We chose to use the Hb value measured between 24 and 30 gestational weeks. If more than one Hb test was performed during those weeks, the mean Hb value was used. This time range was selected since this is the window in which a glucose tolerance test is performed and there is sufficient time for anemia diagnosis and treatment. Consequently, women without a CBC between 24 and 30 gestational weeks, were excluded from the current analysis.

### Study groups

Women included in the present analysis were divided according to the results of the Hb test taken at delivery, to women with (Hb < 10.5 g/dL) and without (Hb ≥ 10.5 g/dL) anemia. Women with mild anemia were included in the anemia group since treatment has clinical benefit in these cases as well.

### Study endpoints

The primary endpoint was to find the cutoff Hb value at 24–30 gestational weeks that can predict anemia at delivery.

Obstetric and demographic characteristics of women with anemia at delivery were also evaluated.

### Statistical analysis

The Hb cutoff to predict anemia at delivery was calculated using the area under the receiver operating characteristic (ROC) curve, as previously described^16,17^.

We wanted to test if the Hb values taken between 24 and 30 gestational weeks had an accuracy of at least 80% to predict anemia at various severities (AUC 80% instead of 70%). We determined a power of 92% with alpha 5%. Also, we assumed that the frequency of anemia will be 12%^14^, requiring a total sample of 800 women. The sample size and power calculations were performed using SAS %ROCPOWER macro^18^. The optimal cut-off point to discriminate anemia at delivery was determined by calculating the ROC sensitivity and specificity pairs and choosing the pair with the minimal distance between them.

The predictive value of Hb measured in the first trimester and during 30–36 gestational weeks was also assessed using the ROC curve.

Baseline characteristics and outcomes of the study groups were compared using the Student’s *t*-test (or Wilcoxon two sample test) for continuous variables and χ2 (or Fisher's exact test) for categorical variables. Independent risk factors for anemia at delivery were calculated using stepwise multivariable logistic regression.

Spearman’s correlation coefficient was used to determine the correlation between Hb at delivery and ARS score.

LOESS curves were used to determine mean Hb throughout pregnancy for women with frequent compared to infrequent use of iron supplements. To this end, we combined into one group, women who reported "never" and "seldom" use of iron supplements (infrequent use) and a second group of women reporting "frequent", "almost always" and "always" use of iron supplements (frequent use). 95% confidence intervals of the LOESS curves are presented as previously described^19^.

Statistical analyses were carried out with SAS version 9.4 (SAS Institute, Cary, NC, USA). Significance was set at a *p* value of < 0.05. Bonferroni correction was performed for multiple comparisons.

## Results

Of the 1,558 women who were included in the original study, 1,527 women delivered at gestational week ≥ 36 and 887 women had CBC tests performed between gestational weeks 24–30. Women with Hb < 10.5 g/dL at delivery had a lower socioeconomic status, higher rate of primiparity and lower Hb at 24–30 gestational weeks compared to women with Hb ≥ 10.5 g/dL near delivery. Of women with available data on overall use of iron supplements the rate of overall use was similar between the groups, but more women in the Hb < 10.5 g/dL group reported on less frequent use (Table 1).

**Table 1:** Characteristics of pregnant women with versus without anemia at delivery.

	Hb ≥ 10.5 g/dL N = 785	Hb < 10.5 g/dL N = 102	*p*
Age (Y)	29.8 (5.3) [26–34]	28.9 (4.9) [25–32]	0.11
BMI (Kg/M^2^)	23.8 (4.5) [23.1, 20.6–25.9]	23.8 (4.3) [23.0, 20.8–25.8]	0.85
Residency ≥ 20,000 people	346 (44%)	38 (37%)	0.19
Socioeconomic status*	− 0.33 (0.6) [(− 0.19), (− 0.62)–(− 0.05)]	− 0.52 (0.45) [(− 0.25), (− 0.83)–(− 0.19)]	0.001
Number of deliveries	2.3 (1.4) [2,1–3]	2.6 (1.3) [2, 2–3]	0.009
Primiparity	268 (34%)	20 (20%)	0.003
Beta thalassemia minor	5 (0.6%)	6 (6%)	< .0001
Delivery week	39.5 (1.2) [39.6,38.6–40.3]	39.3 (1.2) [39.3,38.4–40.3]	0.33
**Ethnicity**			
Jews	512 (65%)	57 (56%)	0.06
Arabs	252 (32%)	44 (43%)	
Other	21 (3%)	1 (1%)	
GDM	83 (11%)	12 (12%)	0.71
Pre-GDM	7 (1%)	0 (0%)	1
Chronic hypertension	10 (1%)	0 (0%)	0.61
Gestational hypertension	24 (3%)	0 (0%)	0.1
Vegetarianism**	40 (7%)	6 (8%)	0.62
Oral iron supplements**	510 (92%)	65 (93%)	0.85
Oral iron supplements frequency**			
Never	43 (8%)	5 (7%)	0.001
Seldom	64 (12%)	19 (28%)	
Frequent	79 (14%)	13 (19%)	
Almost always	104 (19%)	14 (20%)	
Always	258 (47%)	18 (26%)	
IV iron sucrose treatment	18 (2.3%)	7 (7.5%)	0.005
Hb at 24–30 weeks	11.1 (0.9) [11.2, 10.6–11.7]	10.1 (0.9) [10.2, 9.6–10.7]	< 0.0001

Values are presented as median [Interquartile range] or number (percent).

*Socioeconomic status was ranked using the 2015 Socio-Economic index of the local municipalities, which is published by the Israeli Central Bureau of Statistics. The index of 2015 is available at: URL: https://www.cbs.gov.il/he/mediarelease/doclib/2018/351/24_18_351t1.pdf.

The index is calculated by means of factor analysis, and is standardized so that the mean index value for all the local authorities is zero. The index value is the distance of the local authority from the mean value measured by standard deviation units. This index was not available for very small authorities (N = 249).

**Data regarding iron supplements, iron supplements frequency, vegetarianism and IV iron sucrose treatment were missing for 264, 270, 215 and 32 women who did not answered these questions.

BMI, body mass index; GDM, gestational diabetes mellitus; IV, intravenous.

After controlling for socioeconomic status, primiparity, delivery number, oral and intravenous iron supplement use and Hb at 24–30 gestational weeks, only socioeconomic status, Hb at 24–30 weeks and seldom versus always iron supplement use remained statistically significant (adjusted OR 0.3 95%CI [0.19–0.45], OR 0.5 95%CI [0.23–0.96] and OR 4.8 95%CI [1.7–13.3] ; respectively, C statistics 84%).

Independent risk factors for anemia at delivery were sought by using stepwise multivariable logistic regression. We incorporated all the characteristics described in Table 1 that occurred at a rate of at least 3% of the population. Iron supplement use was divided to frequent and infrequent use as described in the Methods section.

Hb at 24–30 gestational weeks and infrequent iron supplement use were independent risk factors for Hb < 10.5 g/dL at delivery (Hb OR 0.3 95%CI [0.2–0.4] and infrequent use of iron supplements OR 2.4 95%CI [1.2–4.8]; C statistics 83%).

### Finding the cutoff Hb value at 24–30 gestational weeks that can predict anemia at delivery

The prediction accuracy of Hb measured between 24 and 30 gestational weeks, to predict anemia at delivery was evaluated using the ROC curve. Hb taken during this time range was accurate to predict anemia at delivery of Hb < 10.5, Hb < 10 and Hb < 9.5 g/dL (AUC 80–85% 95%CI 75–94%; Table 2). Hb < 10.6 g/dL was the best predictor for all the mentioned Hb values at delivery, with 75% sensitivity and 74% specificity in predicting Hb < 10.5 g/dL at delivery (Table 2). First-trimester Hb level was a much weaker predictor for anemia at delivery (AUC 70% 95%CI 64–76%).

**Table 2:** Prenatal anemia prediction accuracy of Hb < 10.6 g/dL between 24 and 30 gestational week to predict anemia before delivery using ROC curve analysis.

Hb before delivery (g/dL)	AUC	95% Confidence intervals	Sensitivity	Specificity
< 10.5	80%	75–84%	75%	74%
< 10	85%	80–90%	86%	75%
< 9.5	85%	76–94%	91%	70%

ROC curve analysis was used to predict anemia at delivery.

AUC, area under the curve; ROC, receiver operating characteristic.

**Figure 1:**
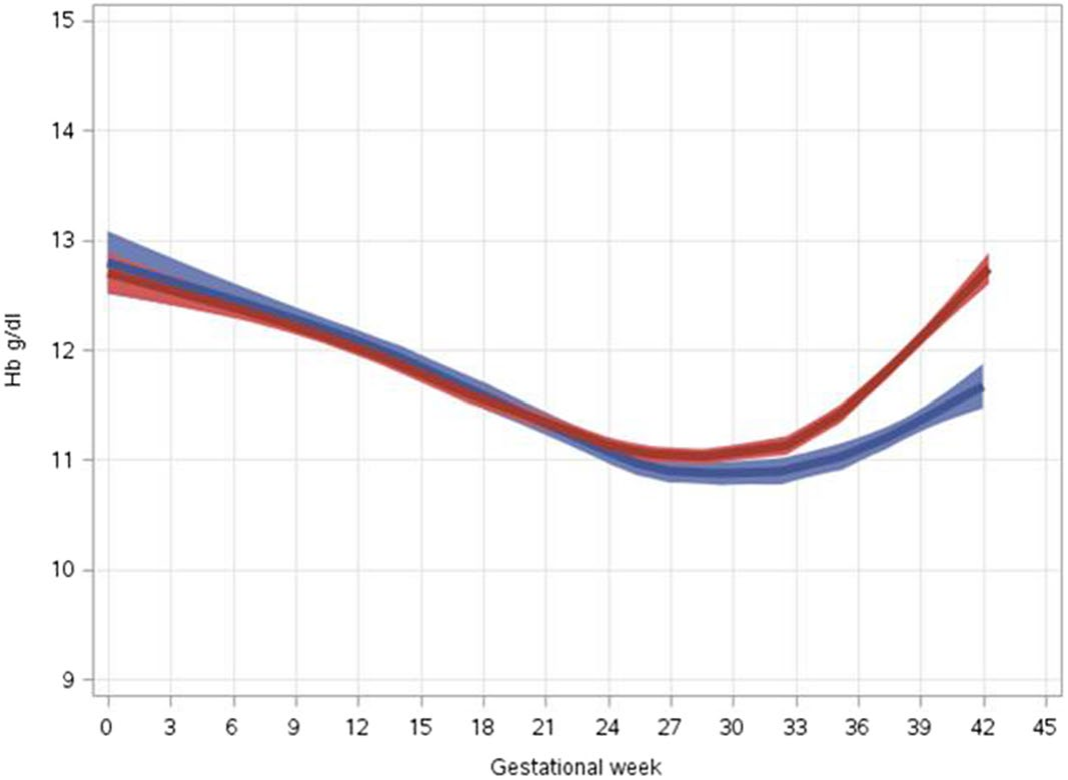
Hemoglobin throughout pregnancy. LOESS curve of hemoglobin levels among pregnant women with frequent (red line) and infrequent (blue line) use of iron supplements.

We used the LOESS curve to assess mean Hb throughout pregnancy for women with frequent and infrequent use of iron supplements (3,939 Hb tests; Fig. 1). Until gestational week 24, Hb values dropped in both groups to a similar level. However, from around gestational week 30, women frequently using iron supplements had a higher increase in Hb level and reached higher Hb at delivery as compared to women with infrequent use.

Women who had infrequent use of iron supplements had twice the risk for anemia at delivery (24/131 (18%) versus 45/486 (9%), *p* = 0.004).

In a subanalysis of women categorized according to Hb level between weeks 24–30 (≤ 10.6 g/dL) and iron supplementation use (frequent versus infrequent; Table 3), women with Hb < 10.6 g/dL showed a two-fold higher risk for anemia at delivery when using iron supplements infrequently as compared to those reporting frequent use of iron supplements (44% versus 25%, *p* = 0.03). In women with Hb ≥ 10.6 g/dL, risk for anemia at delivery was 10% when iron supplements were used infrequently, while the risk in frequent users reached only 3% (*p* = 0.005; Table 3).

**Table 3:** Rate of anemia at delivery (Hb < 10.5 g/dL) according to Hb level at 24–30 weeks and iron supplementation.

Iron supplementation	Hb at 24–30 weeks		
	Hb < 10.6 g/dL	Hb ≥ 10.6 g/dL	*p*
Infrequent	14/32 (44%)	10/99 (10%)	< 0.0001
Frequent	35/142 (25%)	10/344 (3%)	< 0.0001
p	0.03	0.005	

This table addresses the 617 women who responded the question regarding iron supplementation use during pregnancy.

Hb, hemoglobin.

ARS scores were available for 693 women. Hb value at delivery weakly and negatively correlated with ARS score (Spearman’s correlation coefficient − 0.2, *p* < 0.0001), suggesting that symptoms related to anemia are not good predictors of anemia.

### Assessing the risk for anemia at delivery according to Hb measured at gestational weeks 30–36

To assess whether Hb measured at 30–36 weeks could predict anemia at delivery, the data from all women who delivered ≥ 36 gestational weeks were analyzed. Using the AUC of the ROC curve, we found that Hb was a strong predictor for anemia at delivery (AUC 90% 95%CI 87–93%). Hb < 10.6 g/dL at 30–36 weeks predicted a risk for anemia at delivery with 89% sensitivity and 76% specificity.

## Discussion

In the present study, we aimed to identify predictive characteristics of pregnant women for anemia at delivery. We found that Hb taken at 24–30 gestational weeks as well as infrequent iron supplement use were independent risk factors for anemia at delivery and Hb < 10.6 g/dL was a good predictor for anemia at delivery (Hb < 10.5, Hb < 10 and Hb < 9.5 g/dL). The use of iron supplements decreased the rate of anemia at delivery by half regardless of Hb value during pregnancy and thus, this treatment should be recommended even for women without anemia.

Iron deficiency is the leading cause of gestational anemia. A pre-pregnancy store of more than 500 mg of iron is required to fulfill the demand of both the mother and fetus and to avoid iron deficiency during pregnancy. However, such levels of iron are only present in 20% of menstruating women before pregnancy^20^. Thus, routine administration of iron supplements during pregnancy is recommended^1,21^. Yet, many women develop gestational anemia due to either malabsorption of oral iron supplements or low compliance due to adverse effects of orally administrated iron supplements, mainly gastrointestinal adverse effects^22^. In such cases, intravenous iron supplement is recommended^21,23,24^ and has been shown to be more effective than oral supplements in increasing Hb at delivery, and more tolerable^25^. In the primary analysis of this study^14^, we demonstrated that approximately 50% of women with Hb < 10.5 g/dL at delivery developed moderate to severe postpartum anemia (Hb ≤ 9.5 g/dL), for which intravenous iron sucrose treatment is advised by our departmental protocol and by others^14,23,24^.

**Figure 2:**
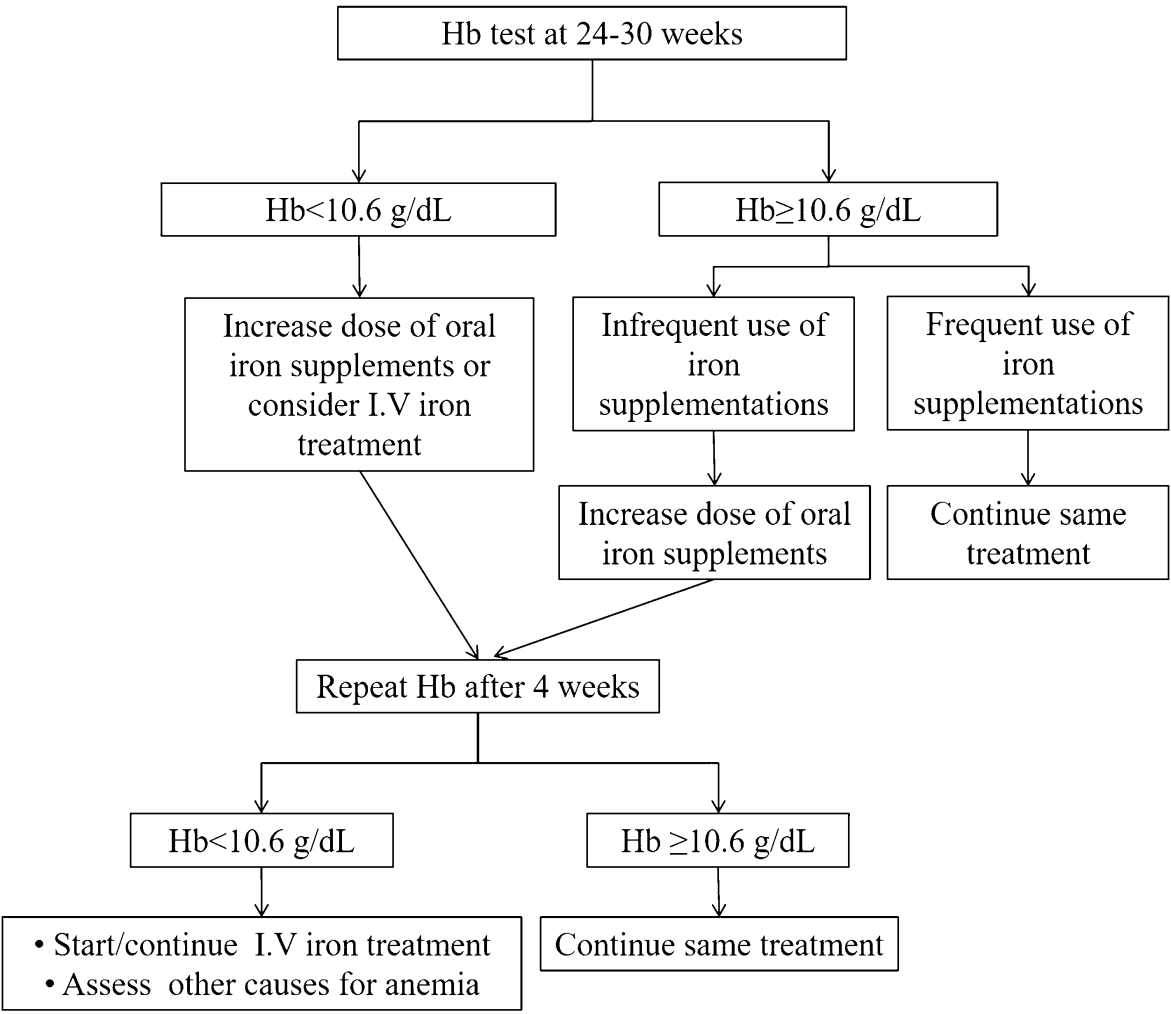
Suggested gestational anemia treatment flow chart based on hemoglobin values and frequency of iron supplementation use. Hb, hemoglobin; I.V intravenous.

To date, there is no consensus with regards to the timing of screening for and how to treat gestational anemia, and particularly how to avoid anemia at delivery. Several official guidelines regarding this critical condition are available in several countries and regions in the world^2,13,23,24^. Yet, it is acknowledged that they are based mainly on expert opinions and personal experience with iron treatment^13,24^. Following the results of the current study, the primary analysis of the original study^14^ as well as previous studies that demonstrated the efficacy of iron supplement use during pregnancy^25,26^, we suggest a flow diagram of screening time and an iron supplement treatment regimen according to Hb levels and the baseline frequency of iron treatment during Hb test (Fig. 2). Screening for anemia between 24 and 30 gestational weeks is appropriate for gestational diabetes diagnosis so both tests could be combined. In our opinion, it is justified to start empiric treatment with iron without the need for additional tests for the following reasons; firstly, iron treatment is a routine recommendation during pregnancy. Secondly, iron deficiency is the leading cause of anemia during pregnancy, and finally, the results of this study demonstrated efficacy of iron treatment regardless of Hb level. However, some clinicians might choose to also use ferritin levels to diagnose iron deficiency as suggested previously^27^ and to use ferritin levels to modify the dose of iron treatment^28^. In this regard, it should be noted that the definition of normal ferritin levels during pregnancy varies between studies and unified international thresholds of iron deficiency for women throughout pregnancy are required^29^. According to both Hb behavior throughout pregnancy (Supplement 1) and the AUC of the ROC curve demonstrating that Hb between 30 and 36 gestational weeks is a strong predictor for anemia at delivery, testing Hb levels at 24–30 weeks and then four weeks later is reasonable. Persistent anemia despite treatment with iron supplements, requires identification of other causes for anemia. In women with Hb ≥ 10.6 g/dL and regular use of iron supplements (56% of the cohort in this study), a follow-up test is not required as only 3% developed anemia at delivery.

The strengths of this study lay in the prospectively collected data, the large sample size, large volume of CBC test results and the analysis of several characteristics that could influence anemia.

The limitations of this study included its nature as a secondary analysis and performance of CBC tests during routine pregnancy follow-up visits. In addition, patient-reported data regarding iron supplements use may have been inaccurate, but is assumed to have been an overestimation of actual use patterns and therefore the effect of iron supplements use is even greater. An additional limitation of this study was the lack of data regarding descriptive analysis of anemia, type of the anemia and iron studies. These elements should be examined while performing an anemia workup and treatment.

## Conclusions

We suggest that Hb levels should be routinely tested between gestational weeks 24–30 to screen for anemia. Hb < 10.6 g/dL during this period is a strong predictor for anemia at delivery and together with the frequency of iron supplement use should dictate the recommended therapy. In case of Hb < 10.6 g/dL or infrequent use of iron supplements, regardless of Hb levels, additional CBC test for anemia should be obtained after four weeks. If anemia is still evident, other causes should be explored and intravenous iron preparations should be administered.

## Supplementary Information

**Supplement 1:** LOESS curve of hemoglobin levels among pregnant women according to gestational week.

Supplementary Information 1.

## Data Availability

Data generated during this study is available upon a reasonable request and with accordance to the regulations of the institutional review board of Emek medical center.
