# Supplementary figures and images for: Prediction of anemia at delivery
Source: Sci Rep. 2021 Mar 18;11:6309. doi: 10.1038/s41598-021-85622-7 (PMC7973554; doi:10.1038/s41598-021-85622-7)

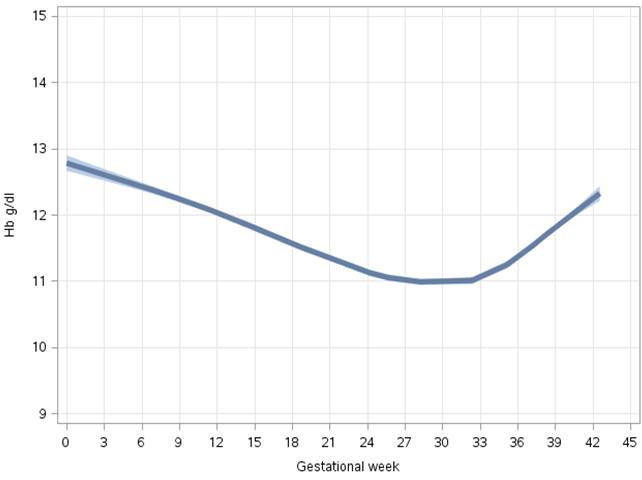

Supplement: Supplementary file 1 — Supplementary Information 1. [file 41598_2021_85622_MOESM1_ESM.jpg]
